# Supplementary material for: Association between hypertension and impaired lung function among adults: A systematic review and meta-analysis
Source: PLoS One. 2026 Apr 10;21(4):e0346569. doi: 10.1371/journal.pone.0346569 (PMC13068241; doi:10.1371/journal.pone.0346569)
Supplement: S1 File — (PDF) [file pone.0346569.s001.pdf]

NIHR | National Institute for Health and Care Research

PROSPERO
International prospective register of systematic reviews

Association between hypertension and impaired lung function among adults: A systematic review and meta-analysis

Dilakshi Lekamge, Anuradhani Kasturiratne, Malay Kanti Mridha, John Chambers

Citation
Dilakshi Lekamge, Anuradhani Kasturiratne, Malay Kanti Mridha, John Chambers. Association between hypertension and impaired lung function among adults: A systematic review and meta-analysis. PROSPERO 2023 CRD42023427631. Available from https://www.crd.york.ac.uk/PROSPERO/view/CRD42023427631.

REVIEW TITLE AND BASIC DETAILS

Review title

Association between hypertension and impaired lung function among adults: A systematic review and meta-analysis

Review objectives

Is there an association between hypertension and impaired lung function among adults?

Keywords

hypertension; impaired lung function; association; Systematic review; meta-analysis; adults

SEARCHING AND SCREENING

Searches 1 change

Sources: Bibliographic databases, reference lists of eligible studies and review articles, and Internet resources. Review articles (literature reviews, systematic reviews, and meta-analyses), qualitative studies, trial registers, case reports, case series, book chapters, letters to editors, protocols, and conference proceedings will be excluded. Databases: MEDLINE (PubMed), Scopus, Web of Science, EMBASE, CINAHL (EBSCOhost). Grey literature will be searched through Google Scholar, ProQuest, and snowballing method, where the references of relevant papers will be hand-searched. Search dates (from and to): Database inception to 22nd July 2025. The searches will be conducted without any language restrictions, and the literature search will be performed to identify relevant studies published from the database inception to 22nd July 2025. The searches will be rerun prior to the final analyses, and any further studies will be identified and retrieved for inclusion. Unpublished studies will be sought(Hypertension OR "high blood pressure" OR "elevated blood pressure" OR "increased blood pressure" OR "raised blood pressure" OR "Hypertens*") AND ("forced expiratory volume" OR "forced vital capacity" OR "timed vital capacity" OR "vital capacity" OR "spirometry" OR "respiratory physiological phenomena*" OR "respiratory physiological phenomenon*" OR "lung pathophysiology" OR "total lung capacit*" OR "impaired lung function" OR "declined lung function" OR "reduced lung function" OR "limited lung function" OR "reduced spirometry" OR

"impaired spirometry" OR "reduced FEV1" OR "reduced forced expiratory volume in 1 second" OR "reduced FVC" OR "reduced forced expiratory volume" OR "reduced VC" OR "reduced forced vital capacity" OR "FEV1/FVC ratio") AND ("adult*" OR "elder*")

Study design

Only nonrandomized study types will be included.

Included

Observational studies including cohort, case-control and cross-sectional studies will only be included.

Excluded

Review articles (literature reviews, systematic reviews, and meta-analyses), qualitative studies, trials registers, case reports, case series, book chapters, letters to editors, protocols, and conference proceedings will be excluded.

Link to search strategy

A full search strategy is not available.

ELIGIBILITY CRITERIA

Condition or domain being studied

HypertensionImpaired lung function

Population

Included

Inclusion: Adult males or females ≥18 years irrespective of their comorbidityExclusion: • Acute respiratory diseases (Example: Bronchitis) • Physical disability that may affect lung function (Scoliosis and Kyphosis) • Patients contraindicated for doing spirometry (Recent myocardial infarction, heart failure) • Surgery to eyes (cataract surgery, glaucoma), chest or abdomen done less than 3 months prior to the study. • Pregnancy

Intervention(s) or exposure(s)

Included

Hypertension and impaired lung functionHypertension will be defined as systolic blood pressure ≥ 140 mmHg and/or diastolic blood pressure ≥ 90 mmHg or use of antihypertensive medication at the time of examination or self-reported history of hypertension or physician diagnosis of hypertension.Hypertension will be considered according to the above definition irrespective of the type of device used (mercury sphygmomanometer or digital blood pressure monitor), time of measuring the blood pressure (daytime, night-time, or continuous basis for 24 hours), the number of blood pressure readings taken (two or three blood pressure readings in succession) and the measurement location (clinic, home or office).Lung function impairment will be assessed based on FVC, FEV1 and FEV1/FVC spirometric measurements.The studies that do not mention the method of measuring lung function will be excluded. The studies based on spirometry to assess lung function will only be selected. No interventions will be considered.

Comparator(s) or control(s)

This review does not have any comparators

Context

Articles in which the definition of hypertension and impaired lung function remain unclear and and studies that do not mention the method of measuring lung function were excluded.

OUTCOMES TO BE ANALYSED

Main outcomes

Association between hypertension and impaired lung function

Additional outcomes

None

DATA COLLECTION PROCESS

Data extraction (selection and coding)

All records identified during the database search will be pooled and the duplicates will be removed using the Zotero reference management software. The PRISMA flow diagram will be used to summarize the selection process. Titles/abstracts of studies retrieved using the search strategy will be screened independently by two reviewers to identify studies that meet the above-mentioned inclusion criteria. The full text of the studies will be independently assessed for eligibility by two reviewers. Disagreements between individual judgments will be resolved through the discussion between reviewers.A data extraction table will be designed with the use of Microsoft Excel to categorize and record the data. The findings of each study will be based on the authors, title, year of publication, study setting (region and country), study design, population, study period, inclusion and exclusion criteria of the study, sample size, gender, age and ethnicity of the participants, relevant variables, statistical method, findings, and limitations. Two reviewers will extract data independently. Any disagreements will be identified and resolved through discussion. Study investigators will be contacted to obtain any unreported data.

Risk of bias (quality) assessment

The full-text articles meeting eligibility will be examined by two review authors independently to assess the risk of bias. The Newcastle-Ottawa Scale (NOS) or Joanna Briggs Institute (JBI) critical appraisal checklist will be used as appropriate tools to assess the quality of included studies. NOS will be used as the evaluation criteria for cohort and case-control studies. For qualitative assessment of the cross-sectional studies, the JBI Critical Appraisal Checklist for cross-sectional studies will be used. Any disagreements will be identified and resolved through discussion.

PLANNED DATA SYNTHESIS

Strategy for data synthesis

The raw data of extraction will be used to compute the pooled odds ratio (OR) estimates with 95% confidence interval (CI) to assess the strength of the association between hypertension and impaired lung function. The heterogeneity across the included studies will be measured using Cochran's Q test and the I^2 statistic. If significant heterogeneity is detected, the random effects models will be applied to estimate pooled effect size. The results will be summarized using forest plots. Potential publication bias will be graphically identified using funnel plots and assessed through Begg's test and Egger's test. Trim and fill method will be used to minimize the effects of publication bias. A meta-regression analysis will also be carried out to detect sources of heterogeneity.Based on the quality assessment, sensitivity analysis may be performed by excluding one study at a time and assessing whether the results are strongly influenced by a single study in order to exclude poor-quality studies.All statistical analyses will be performed using R Studio software.

Analysis of subgroups or subsets

If a statistically significant amount of heterogeneity is detected a subgroup analysis will be carried out to determine how the association between hypertension and impaired lung function differ by the gender, BMI, ethnicity, and other confounding factors.

REVIEW AFFILIATION, FUNDING AND PEER REVIEW

Review team members

Ms Dilakshi Lekamge (review guarantor and contact) ORCID: 0000-0002-7568-5564. Department of Public Health, Faculty of Medicine, University of Kelaniya, Sri Lanka. Sri Lanka.

No conflict of interest declared.

Professor Anuradhani Kasturiratne. Department of Public Health, Faculty of Medicine, University of Kelaniya, Sri Lanka. Sri Lanka.

No conflict of interest declared.

Professor Malay Kanti Mridha. James P Grant School of Public Health, BRAC University, Bangladesh. Bangladesh.

No conflict of interest declared.

Professor John Chambers. Faculty of Medicine, School of Public Health, Imperial College London. England.

No conflict of interest declared.

Named contact

Ms Dilakshi Lekamge (dilakshi.lekamge@gmail.com). ORCID: 0000-0002-7568-5564. Department of Public Health, Faculty of Medicine, University of Kelaniya, Sri Lanka. Sri Lanka.

Review affiliation

Department of Public Health, Faculty of Medicine, University of Kelaniya, Sri Lanka

Funding source

Review has no specific/external funding but is supported by guarantor/review team (non-commercial) institutions.

TIMELINE OF THE REVIEW

Review timeline

Start date: 1 June 2023. End date: 30 August 2025.

Date of first submission to PROSPERO

30 May 2023

Date of registration in PROSPERO

30 May 2023

CURRENT REVIEW STAGE

Publication of review results

Results of the review will be published in English.

Journal publication

Not yet published in a journal but will be in future.

Stage of the review at this submission

Review stage	Started	Completed
Pilot work	✓	✓
Formal searching/study identification	✓	✓
Screening search results against inclusion criteria	✓	✓
Data extraction or receipt of IPD	✓	✓
Risk of bias/quality assessment	✓	✓
Data synthesis	✓	✓

Review status

The review is completed.

ADDITIONAL INFORMATION

PROSPERO version history 1 change

- Version 2.0, published 25 Oct 2025
- Version 1.1, published 02 Oct 2025
- Version 1.0, published 30 May 2023

Review conflict of interest

Declared individual interests are recorded under team member details.. No additional interests are recorded for this review.

Country

Sri Lanka; England; Bangladesh

Medical Subject Headings

Adult; Humans; Hypertension

Revision note 1 change

In the previous version, it was stated under the *Searches* section that "The searches will be restricted to studies published in English." However, during the actual literature search, no language restrictions were applied, and all retrieved records, irrespective of language, were included in the screening process. Therefore, in this version, that sentence has been revised to "The searches will be conducted without any language restrictions."

Disclaimer

The content of this record displays the information provided by the review team. PROSPERO does not peer review registration records or endorse their content.

PROSPERO accepts and posts the information provided in good faith; responsibility for record content rests with the review team. The guarantor for this record has affirmed that the information provided is truthful and that they understand that deliberate provision of inaccurate information may be construed as scientific misconduct.

PROSPERO does not accept any liability for the content provided in this record or for its use. Readers use the information provided in this record at their own risk.

Any enquiries about the record should be referred to the named review contact
