# Supplementary material for: Association between hypertension and impaired lung function among adults: A systematic review and meta-analysis
Source: PLoS One. 2026 Apr 10;21(4):e0346569. doi: 10.1371/journal.pone.0346569 (PMC13068241; doi:10.1371/journal.pone.0346569)
Supplement: S1 Table — (DOCX) [file pone.0346569.s008.docx]

|  | Study Omitted | Summary OR |  | 95% CI | z | p - value | I^2^ | Q | p-value |
| --- | --- | --- | --- | --- | --- | --- | --- | --- | --- |
|  | Nothing Omitted | 1.6957 | 1.70 | [1.5312– 1.8779] | 10.14 | < 0.0001 | 84.2% | 190.42 | < 0.0001 |
| 1 | Bozek et al., 2016 | 1.7331 | 1.73 | [1.5823; 1.8982] | 11.84 | < 0.0001 | 81.4% | 155.93 | < 0.0001 |
| 2 | Bozek et al., 2016 | 1.6947 | 1.69 | [1.5235; 1.8851] | 9.71 | < 0.0001 | 84.8% | 190.33 | < 0.0001 |
| 3 | Dharmage et al., 2023 | 1.7305 | 1.73 | [1.5752; 1.9012] | 11.43 | < 0.0001 | 82.2% | 162.49 | < 0.0001 |
| 4 | Dharmage et al., 2023 | 1.6983 | 1.70 | [1.5275; 1.8881] | 9.80 | < 0.0001 | 84.8% | 190.39 | < 0.0001 |
| 5 | Dharmage et al., 2023 | 1.7120 | 1.71 | [1.5448; 1.8972] | 10.26 | < 0.0001 | 84.5% | 187.49 | < 0.0001 |
| 6 | Di Raimondo et al., 2020 | 1.6832 | 1.68 | [1.5203; 1.8635] | 10.03 | < 0.0001 | 84.6% | 187.91 | < 0.0001 |
| 7 | Ferguson et al., 2014 | 1.6785 | 1.68 | [1.5143; 1.8605] | 9.86 | < 0.0001 | 84.5% | 187.30 | < 0.0001 |
| 8 | Ferguson et al., 2014 | 1.6858 | 1.69 | [1.5197; 1.8700] | 9.87 | < 0.0001 | 84.7% | 189.37 | < 0.0001 |
| 9 | Ferguson et al., 2014 | 1.6710 | 1.67 | [1.5126; 1.8461] | 10.10 | < 0.0001 | 84.3% | 184.22 | < 0.0001 |
| 10 | Ferguson et al., 2014 | 1.6948 | 1.69 | [1.5276; 1.8802] | 9.96 | < 0.0001 | 84.8% | 190.40 | < 0.0001 |
| 11 | Jo et al., 2015 | 1.6972 | 1.70 | [1.5252; 1.8885] | 9.71 | < 0.0001 | 84.8% | 190.42 | < 0.0001 |
| 12 | Kaufmann et al., 2024 | 1.6851 | 1.69 | [1.5162; 1.8729] | 9.68 | < 0.0001 | 84.5% | 187.08 | < 0.0001 |
| 13 | Kiani & Ahmadi, 2021 | 1.7082 | 1.71 | [1.5379; 1.8974] | 9.99 | < 0.0001 | 84.6% | 188.62 | < 0.0001 |
| 14 | Kim et al., 2017 | 1.7003 | 1.70 | [1.5285; 1.8914] | 9.77 | < 0.0001 | 84.8% | 190.19 | < 0.0001 |
| 15 | Lindberg et al., 2011 | 1.6979 | 1.70 | [1.5274; 1.8875] | 9.80 | < 0.0001 | 84.8% | 190.4 | < 0.0001 |
| 16 | Lindberg et al., 2011 | 1.7186 | 1.72 | [1.5512; 1.9041] | 10.35 | < 0.0001 | 84.0% | 180.78 | < 0.0001 |
| 17 | Lindberg et al., 2011 | 1.7094 | 1.71 | [1.5387; 1.8991] | 9.99 | < 0.0001 | 84.5% | 187.68 | < 0.0001 |
| 18 | Mannino et al., 2008 | 1.6950 | 1.69 | [1.5228; 1.8866] | 9.66 | < 0.0001 | 84.7% | 190.06 | < 0.0001 |
| 19 | Mannino et al., 2008 | 1.7172 | 1.72 | [1.5476; 1.9054] | 10.19 | < 0.0001 | 80.5% | 148.81 | < 0.0001 |
| 20 | Mannino et al., 2008 | 1.7072 | 1.71 | [1.5346; 1.8991] | 9.84 | < 0.0001 | 84.1% | 182.84 | < 0.0001 |
| 21 | Mannino et al., 2008 | 1.6844 | 1.68 | [1.5158; 1.8716] | 9.69 | < 0.0001 | 84.5% | 187.08 | < 0.0001 |
| 22 | Mannino et al.2012 | 1.6775 | 1.68 | [1.5116; 1.8616] | 9.74 | < 0.0001 | 83.7% | 178.12 | < 0.0001 |
| 23 | Mannino et al.2012 | 1.6827 | 1.68 | [1.5144; 1.8698] | 9.68 | < 0.0001 | 84.1% | 182.20 | < 0.0001 |
| 24 | Methvin et al., 2009 | 1.6754 | 1.68 | [1.5143; 1.8536] | 10.01 | < 0.0001 | 84.4% | 186.30 | < 0.0001 |
| 25 | Methvin et al., 2009 | 1.6964 | 1.70 | [1.5299; 1.8811] | 10.03 | < 0.0001 | 84.8% | 190.42 | < 0.0001 |
| 26 | Methvin et al., 2009 | 1.6968 | 1.70 | [1.5293; 1.8827] | 9.97 | < 0.0001 | 84.8% | 190.42 | < 0.0001 |
| 27 | Methvin et al., 2009 | 1.6963 | 1.70 | [1.5301; 1.8806] | 10.05 | < 0.0001 | 84.8% | 190.42 | < 0.0001 |
| 28 | Park et al., 2015 | 1.6835 | 1.68 | [1.5147; 1.8711] | 9.66 | < 0.0001 | 83.8% | 179.54 | < 0.0001 |
| 29 | Park et al., 2015 | 1.6917 | 1.69 | [1.5202; 1.8824] | 9.64 | < 0.0001 | 84.6% | 188.32 | < 0.0001 |
| 30 | Sperandio et al., 2016 | 1.6877 | 1.69 | [1.5230; 1.8701] | 9.99 | < 0.0001 | 84.7% | 189.45 | < 0.0001 |
| 31 | Yang et al., 2020 | 1.6816 | 1.69 | [1.5136; 1.8683] | 9.68 | < 0.0001 | 83.6% | 176.79 | < 0.0001 |
| **Removing the whole study at once** | | | | | | | | | |
| 32 | Bozek et al., 2016 | 1.7334 | 1.73 | [1.5758; 1.9068] | 11.31 | < 0.0001 | 82.0% | 155.90 | < 0.0001 |
| 33 | Dharmage et al., 2023 | 1.7517 | 1.75 | [1.5880; 1.9322] | 11.20 | < 0.0001 | 83.1% | 159.34 | < 0.0001 |
| 34 | Di Raimondo et al., 2020 | 1.6832 | 1.68 | [1.5203; 1.8635] | 10.03 | < 0.0001 | 84.6% | 187.91 | < 0.0001 |
| 35 | Ferguson et al., 2014 | 1.6401 | 1.64 | [1.4787; 1.8192] | 9.36 | < 0.0001 | 85.6% | 179.94 | < 0.0001 |
| 36 | Jo et al., 2015 | 1.6972 | 1.70 | [1.5252; 1.8885] | 9.71 | < 0.0001 | 84.8% | 190.42 | < 0.0001 |
| 37 | Kaufmann et al., 2024 | 1.6851 | 1.69 | [1.5162; 1.8729] | 9.68 | < 0.0001 | 84.5% | 187.08 | < 0.0001 |
| 38 | Kiani & Ahmadi, 2021 | 1.7082 | 1.71 | [1.5379; 1.8974] | 9.99 | < 0.0001 | 84.6% | 188.62 | < 0.0001 |
| 39 | Kim et al., 2017 | 1.7003 | 1.70 | [1.5285; 1.8914] | 9.77 | < 0.0001 | 84.8% | 190.19 | < 0.0001 |
| 40 | Lindberg et al., 2011 | 1.7379 | 1.74 | [1.5569; 1.9398] | 9.85 | < 0.0001 | 84.8% | 177.83 | < 0.0001 |
| 41 | Mannino et al., 2008 | 1.7197 | 1.72 | [1.5261; 1.9378] | 8.90 | < 0.0001 | 80.7% | 134.50 | < 0.0001 |
| 42 | Mannino et al.2012 | 1.6631 | 1.66 | [1.4936; 1.8519] | 9.27 | < 0.0001 | 83.4% | 168.62 | < 0.0001 |
| 43 | Methvin et al., 2009 | 1.6766 | 1.68 | [1.5089; 1.8630] | 9.61 | < 0.0001 | 86.0% | 186.29 | < 0.0001 |
| 44 | Park et al., 2015 | 1.6789 | 1.68 | [1.5028; 1.8756] | 9.17 | < 0.0001 | 84.1% | 176.23 | < 0.0001 |
| 45 | Sperandio et al., 2016 | 1.6877 | 1.69 | [1.5230; 1.8701] | 9.99 | < 0.0001 | 84.7% | 189.45 | < 0.0001 |
| 46 | Yang et al., 2020 | 1.6816 | 1.68 | [1.5136; 1.8683] | 9.68 | < 0.0001 | 83.6% | 176.79 | < 0.0001 |
| **Exclusion of studies with low quality or high risk of bias** | | | | | | | | | |
| 47 | Dharmage et al., 2023  Kaufmann et al., 2024  Park et al., 2015 | 1.7287 | 1.73 | [1.5434; 1.9363] | 9.46 | < 0.0001 | 83.3% | 143.95 | < 0.0001 |

**S1 Table. Results of the sensitivity analysis - ILF (Exposure) and HT (Outcome) – Unadjusted analysis**
