# Supplementary material for: Association between hypertension and impaired lung function among adults: A systematic review and meta-analysis
Source: PLoS One. 2026 Apr 10;21(4):e0346569. doi: 10.1371/journal.pone.0346569 (PMC13068241; doi:10.1371/journal.pone.0346569)
Supplement: S2 Table — (DOCX) [file pone.0346569.s009.docx]

**S2 Table. Results of the sensitivity analysis - ILF (Exposure) and HT (Outcome) – Adjusted analysis**

|  | Study Omitted | Summary OR |  | 95% CI | z | p - value | I^2^ | Q | p-value |
| --- | --- | --- | --- | --- | --- | --- | --- | --- | --- |
|  | Nothing Omitted | 1.3986 | 1.40 | [1.3107; 1.4925] | 10.12 | < 0.0001 | 59.8% | 211.70 | < 0.0001 |
| 1 | Di Raimondo et al., 2020 | 1.3943 | 1.39 | [1.3067; 1.4877] | 10.04 | < 0.0001 | 59.7% | 208.53 | < 0.0001 |
| 2 | Ferguson et al., 2014 | 1.3968 | 1.40 | [1.3082; 1.4915] | 9.99 | < 0.0001 | 60.3% | 211.44 | < 0.0001 |
| 3 | Ferguson et al., 2014 | 1.3931 | 1.39 | [1.3057; 1.4865] | 10.02 | < 0.0001 | 59.7% | 208.54 | < 0.0001 |
| 4 | Ferguson et al., 2014 | 1.4015 | 1.40 | [1.3130; 1.4958] | 10.15 | < 0.0001 | 60.2% | 210.92 | < 0.0001 |
| 5 | Jo et al., 2015 | 1.3895 | 1.39 | [1.3019; 1.4831] | 9.90 | < 0.0001 | 58.8% | 203.69 | < 0.0001 |
| 6 | Kiani & Ahmadi, 2021 | 1.3979 | 1.40 | [1.3084; 1.4935] | 9.92 | < 0.0001 | 60.3% | 211.65 | < 0.0001 |
| 7 | Kim et al., 2017 | 1.3921 | 1.39 | [1.3034; 1.4869] | 9.85 | < 0.0001 | 59.6% | 207.95 | < 0.0001 |
| 8 | Kulbacka-Ortiz et al., 2022 | 1.3928 | 1.39 | [1.3048; 1.4867] | 9.95 | < 0.0001 | 59.9% | 209.38 | < 0.0001 |
| 9 | Kulbacka-Ortiz et al., 2022 | 1.3981 | 1.40 | [1.3089; 1.4935] | 9.95 | < 0.0001 | 60.3% | 211.68 | < 0.0001 |
| 10 | Kulbacka-Ortiz et al., 2022 | 1.4019 | 1.40 | [1.3132; 1.4965] | 10.14 | < 0.0001 | 60.2% | 210.93 | < 0.0001 |
| 11 | Kulbacka-Ortiz et al., 2022 | 1.3949 | 1.39 | [1.3066; 1.4892] | 9.97 | < 0.0001 | 60.1% | 210.64 | < 0.0001 |
| 12 | Kulbacka-Ortiz et al., 2022 | 1.4015 | 1.40 | [1.3119; 1.4971] | 10.02 | < 0.0001 | 60.2% | 211.27 | < 0.0001 |
| 13 | Kulbacka-Ortiz et al., 2022 | 1.3933 | 1.39 | [1.3048; 1.4877] | 9.91 | < 0.0001 | 60.0% | 209.79 | < 0.0001 |
| 14 | Kulbacka-Ortiz et al., 2022 | 1.4019 | 1.40 | [1.3129; 1.4969] | 10.10 | < 0.0001 | 60.2% | 211.08 | < 0.0001 |
| 15 | Kulbacka-Ortiz et al., 2022 | 1.3997 | 1.40 | [1.3105; 1.4949] | 10.01 | < 0.0001 | 60.3% | 211.65 | < 0.0001 |
| 16 | Kulbacka-Ortiz et al., 2022 | 1.3967 | 1.40 | [1.3078; 1.4917] | 9.96 | < 0.0001 | 60.3% | 211.45 | < 0.0001 |
| 17 | Kulbacka-Ortiz et al., 2022 | 1.3953 | 1.40 | [1.3069; 1.4897] | 9.98 | < 0.0001 | 60.2% | 210.87 | < 0.0001 |
| 18 | Kulbacka-Ortiz et al., 2022 | 1.4001 | 1.40 | [1.3114; 1.4948] | 10.08 | < 0.0001 | 60.3% | 211.54 | < 0.0001 |
| 19 | Kulbacka-Ortiz et al., 2022 | 1.3953 | 1.40 | [1.3071; 1.4895] | 10.00 | < 0.0001 | 60.1% | 210.73 | < 0.0001 |
| 20 | Kulbacka-Ortiz et al., 2022 | 1.3924 | 1.39 | [1.3045; 1.4861] | 9.96 | < 0.0001 | 59.8% | 209.00 | < 0.0001 |
| 21 | Kulbacka-Ortiz et al., 2022 | 1.3941 | 1.39 | [1.3060; 1.4882] | 9.97 | < 0.0001 | 60.0% | 210.17 | < 0.0001 |
| 22 | Kulbacka-Ortiz et al., 2022 | 1.3954 | 1.40 | [1.3067; 1.4901] | 9.94 | < 0.0001 | 60.2% | 210.98 | < 0.0001 |
| 23 | Kulbacka-Ortiz et al., 2022 | 1.3971 | 1.40 | [1.3085; 1.4917] | 10.01 | < 0.0001 | 60.3% | 211.50 | < 0.0001 |
| 24 | Kulbacka-Ortiz et al., 2022 | 1.3980 | 1.40 | [1.3083; 1.4938] | 9.90 | < 0.0001 | 60.3% | 211.66 | < 0.0001 |
| 25 | Kulbacka-Ortiz et al., 2022 | 1.3931 | 1.39 | [1.3050; 1.4871] | 9.95 | < 0.0001 | 59.9% | 209.56 | < 0.0001 |
| 26 | Kulbacka-Ortiz et al., 2022 | 1.3979 | 1.40 | [1.3093; 1.4924] | 10.03 | < 0.0001 | 60.3% | 211.64 | < 0.0001 |
| 27 | Kulbacka-Ortiz et al., 2022 | 1.3923 | 1.39 | [1.3053; 1.4851] | 10.05 | < 0.0001 | 59.2% | 206.12 | < 0.0001 |
| 28 | Kulbacka-Ortiz et al., 2022 | 1.3981 | 1.40 | [1.3088; 1.4935] | 9.95 | < 0.0001 | 60.3% | 211.67 | < 0.0001 |
| 29 | Kulbacka-Ortiz et al., 2022 | 1.3962 | 1.40 | [1.3077; 1.4908] | 9.98 | < 0.0001 | 60.2% | 211.25 | < 0.0001 |
| 30 | Kulbacka-Ortiz et al., 2022 | 1.3989 | 1.40 | [1.3096; 1.4942] | 9.98 | < 0.0001 | 60.3% | 211.70 | < 0.0001 |
| 31 | Kulbacka-Ortiz et al., 2022 | 1.3914 | 1.39 | [1.3042; 1.4845] | 10.00 | < 0.0001 | 59.6% | 207.78 | < 0.0001 |
| 32 | Kulbacka-Ortiz et al., 2022 | 1.4030 | 1.40 | [1.3139; 1.4982] | 10.11 | < 0.0001 | 60.1% | 210.66 | < 0.0001 |
| 33 | Kulbacka-Ortiz et al., 2022 | 1.4063 | 1.41 | [1.3182; 1.5004] | 10.32 | < 0.0001 | 59.7% | 208.33 | < 0.0001 |
| 34 | Kulbacka-Ortiz et al., 2022 | 1.3966 | 1.40 | [1.3079; 1.4912] | 9.98 | < 0.0001 | 60.3% | 211.37 | < 0.0001 |
| 35 | Kulbacka-Ortiz et al., 2022 | 1.4003 | 1.40 | [1.3117; 1.4949] | 10.09 | < 0.0001 | 60.3% | 211.47 | < 0.0001 |
| 36 | Kulbacka-Ortiz et al., 2022 | 1.3976 | 1.40 | [1.3090; 1.4921] | 10.02 | < 0.0001 | 60.3% | 211.59 | < 0.0001 |
| 37 | Kulbacka-Ortiz et al., 2022 | 1.3955 | 1.40 | [1.3059; 1.4912] | 9.85 | < 0.0001 | 60.2% | 210.87 | < 0.0001 |
| 38 | Kulbacka-Ortiz et al., 2022 | 1.3973 | 1.40 | [1.3087; 1.4919] | 10.01 | < 0.0001 | 60.3% | 211.54 | < 0.0001 |
| 39 | Kulbacka-Ortiz et al., 2022 | 1.3995 | 1.40 | [1.3108; 1.4941] | 10.06 | < 0.0001 | 60.3% | 211.65 | < 0.0001 |
| 40 | Lindberg et al., 2011 | 1.3991 | 1.40 | [1.3095; 1.4948] | 9.95 | < 0.0001 | 60.3% | 211.69 | < 0.0001 |
| 41 | Lindberg et al., 2011 | 1.4016 | 1.40 | [1.3117; 1.4976] | 9.98 | < 0.0001 | 60.2% | 211.16 | < 0.0001 |
| 42 | Lindberg et al., 2011 | 1.3981 | 1.40 | [1.3080; 1.4944] | 9.86 | < 0.0001 | 60.3% | 211.65 | < 0.0001 |
| 43 | Mannino et al., 2008 | 1.3956 | 1.40 | [1.3053; 1.4920] | 9.77 | < 0.0001 | 59.7% | 208.41 | < 0.0001 |
| 44 | Mannino et al., 2008 | 1.4080 | 1.41 | [1.3190; 1.5030] | 10.27 | < 0.0001 | 58.4% | 201.73 | < 0.0001 |
| 45 | Mannino et al., 2008 | 1.3983 | 1.40 | [1.3077; 1.4951] | 9.82 | < 0.0001 | 60.3% | 211.60 | < 0.0001 |
| 46 | Mannino et al., 2008 | 1.3939 | 1.39 | [1.3044; 1.4894] | 9.81 | < 0.0001 | 59.9% | 209.26 | < 0.0001 |
| 47 | Mannino et al.2012 | 1.3835 | 1.38 | [1.3000; 1.4724] | 10.22 | < 0.0001 | 53.2% | 179.54 | < 0.0001 |
| 48 | Mannino et al.2012 | 1.3972 | 1.40 | [1.3069; 1.4938] | 9.81 | < 0.0001 | 60.2% | 211.30 | < 0.0001 |
| 49 | Methvin et al., 2009 | 1.3920 | 1.39 | [1.3048; 1.4850] | 10.02 | < 0.0001 | 59.5% | 207.65 | < 0.0001 |
| 50 | Methvin et al., 2009 | 1.3993 | 1.40 | [1.3110; 1.4935] | 10.10 | < 0.0001 | 60.3% | 211.64 | < 0.0001 |
| 51 | Methvin et al., 2009 | 1.3974 | 1.40 | [1.3090; 1.4918] | 10.03 | < 0.0001 | 60.3% | 211.54 | < 0.0001 |
| 52 | Methvin et al., 2009 | 1.3979 | 1.40 | [1.3096; 1.4921] | 10.07 | < 0.0001 | 60.3% | 211.61 | < 0.0001 |
| 53 | Sperandio et al., 2016 | 1.3960 | 1.40 | [1.3083; 1.4896] | 10.08 | < 0.0001 | 59.5% | 207.23 | < 0.0001 |
| 54 | Triest et al., 2019 | 1.4046 | 1.40 | [1.3165; 1.4987] | 10.28 | < 0.0001 | 59.8% | 208.85 | < 0.0001 |
| 55 | Triest et al., 2019 | 1.3923 | 1.39 | [1.3049; 1.4855] | 10.01 | < 0.0001 | 59.7% | 208.21 | < 0.0001 |
| 56 | Triest et al., 2019 | 1.4033 | 1.40 | [1.3147; 1.4978] | 10.18 | < 0.0001 | 60.1% | 210.31 | < 0.0001 |
| 57 | Triest et al., 2019 | 1.4033 | 1.40 | [1.3154; 1.4971] | 10.26 | < 0.0001 | 59.4% | 207.10 | < 0.0001 |
| 58 | Triest et al., 2019 | 1.4108 | 1.41 | [1.3240; 1.5032] | 10.63 | < 0.0001 | 58.8% | 203.80 | < 0.0001 |
| 59 | Triest et al., 2019 | 1.3991 | 1.40 | [1.3102; 1.4940] | 10.03 | < 0.0001 | 60.3% | 211.69 | < 0.0001 |
| 60 | Triest et al., 2019 | 1.4014 | 1.40 | [1.3130; 1.4959] | 10.14 | < 0.0001 | 60.2% | 210.98 | < 0.0001 |
| 61 | Triest et al., 2019 | 1.4041 | 1.40 | [1.3158; 1.4984] | 10.24 | < 0.0001 | 59.9% | 209.51 | < 0.0001 |
| 62 | Triest et al., 2019 | 1.4033 | 1.40 | [1.3146; 1.4981] | 10.17 | < 0.0001 | 60.1% | 210.40 | < 0.0001 |
| 63 | Triest et al., 2019 | 1.3990 | 1.40 | [1.3106; 1.4934] | 10.08 | < 0.0001 | 60.3% | 211.68 | < 0.0001 |
| 64 | Triest et al., 2019 | 1.3976 | 1.40 | [1.3090; 1.4921] | 10.02 | < 0.0001 | 60.3% | 211.60 | < 0.0001 |
| 65 | Triest et al., 2019 | 1.4062 | 1.41 | [1.3183; 1.4999] | 10.35 | < 0.0001 | 59.6% | 207.73 | < 0.0001 |
| 66 | Triest et al., 2019 | 1.4035 | 1.40 | [1.3149; 1.4980] | 10.20 | < 0.0001 | 60.0% | 210.15 | < 0.0001 |
| 67 | Triest et al., 2019 | 1.4079 | 1.41 | [1.3191; 1.5027] | 10.29 | < 0.0001 | 59.0% | 204.71 | < 0.0001 |
| 68 | Triest et al., 2019 | 1.4097 | 1.41 | [1.3229; 1.5023] | 10.59 | < 0.0001 | 58.8% | 203.98 | < 0.0001 |
| 69 | Triest et al., 2019 | 1.3949 | 1.39 | [1.3065; 1.4892] | 9.97 | < 0.0001 | 60.1% | 210.66 | < 0.0001 |
| 70 | Triest et al., 2019 | 1.4015 | 1.40 | [1.3127; 1.4964] | 10.10 | < 0.0001 | 60.2% | 211.17 | < 0.0001 |
| 71 | Triest et al., 2019 | 1.4025 | 1.40 | [1.3143; 1.4965] | 10.21 | < 0.0001 | 59.9% | 209.67 | < 0.0001 |
| 72 | Triest et al., 2019 | 1.4011 | 1.40 | [1.3128; 1.4953] | 10.16 | < 0.0001 | 60.2% | 210.87 | < 0.0001 |
| 73 | Triest et al., 2019 | 1.3960 | 1.40 | [1.3081; 1.4899] | 10.05 | < 0.0001 | 60.1% | 210.49 | < 0.0001 |
| 74 | Triest et al., 2019 | 1.4021 | 1.40 | [1.3120; 1.4985] | 9.97 | < 0.0001 | 60.1% | 210.59 | < 0.0001 |
| 75 | Triest et al., 2019 | 1.4033 | 1.40 | [1.3150; 1.4975] | 10.22 | < 0.0001 | 60.0% | 209.81 | < 0.0001 |
| 76 | Triest et al., 2019 | 1.4020 | 1.40 | [1.3137; 1.4961] | 10.19 | < 0.0001 | 60.0% | 210.21 | < 0.0001 |
| 77 | Triest et al., 2019 | 1.3987 | 1.40 | [1.3098; 1.4936] | 10.02 | < 0.0001 | 60.3% | 211.70 | < 0.0001 |
| 78 | Triest et al., 2019 | 1.3986 | 1.40 | [1.3103; 1.4928] | 10.09 | < 0.0001 | 60.3% | 211.70 | < 0.0001 |
| 79 | Triest et al., 2019 | 1.4029 | 1.40 | [1.3137; 1.4982] | 10.10 | < 0.0001 | 60.1% | 210.73 | < 0.0001 |
| 80 | Triest et al., 2019 | 1.3919 | 1.39 | [1.3045; 1.4851] | 10.00 | < 0.0001 | 59.6% | 208.16 | < 0.0001 |
| 81 | Triest et al., 2019 | 1.4057 | 1.41 | [1.3179; 1.4992] | 10.36 | < 0.0001 | 59.3% | 206.60 | < 0.0001 |
| 82 | Triest et al., 2019 | 1.4036 | 1.40 | [1.3153; 1.4977] | 10.24 | < 0.0001 | 59.9% | 209.43 | < 0.0001 |
| 83 | Triest et al., 2019 | 1.3851 | 1.39 | [1.3015; 1.4740] | 10.26 | < 0.0001 | 57.6% | 198.09 | < 0.0001 |
| 84 | Triest et al., 2019 | 1.4009 | 1.40 | [1.3123; 1.4955] | 10.11 | < 0.0001 | 60.2% | 211.29 | < 0.0001 |
| 85 | Triest et al., 2019 | 1.3999 | 1.40 | [1.3110; 1.4948] | 10.05 | < 0.0001 | 60.3% | 211.60 | < 0.0001 |
| 86 | Yang et al., 2020 | 1.4086 | 1.41 | [1.3196; 1.5036] | 10.29 | < 0.0001 | 54.0% | 182.71 | < 0.0001 |
| **Removing the whole study at once** | | | | | | | | | |
| 87 | Di Raimondo et al., 2020 | 1.3943 | 1.39 | [1.3067; 1.4877] | 10.04 | < 0.0001 | 59.7% | 208.53 | < 0.0001 |
| 88 | Ferguson et al., 2014 | 1.3941 | 1.39 | [1.3055; 1.4887] | 9.92 | < 0.0001 | 60.5% | 207.51 | < 0.0001 |
| 89 | Jo et al., 2015 | 1.3895 | 1.39 | [1.3019; 1.4831] | 9.90 | < 0.0001 | 58.8% | 203.69 | < 0.0001 |
| 90 | Kiani & Ahmadi, 2021 | 1.3979 | 1.40 | [1.3084; 1.4935] | 9.92 | < 0.0001 | 60.3% | 211.65 | < 0.0001 |
| 91 | Kim et al., 2017 | 1.3921 | 1.39 | [1.3034; 1.4869] | 9.85 | < 0.0001 | 59.6% | 207.95 | < 0.0001 |
| 92 | Kulbacka-Ortiz et al., 2022 | 1.3242 | 1.32 | [1.2047; 1.4556] | 5.82 | < 0.0001 | 70.2% | 178.15 | < 0.0001 |
| 93 | Lindberg et al., 2011 | 1.4016 | 1.40 | [1.3077; 1.5022] | 9.54 | < 0.0001 | 61.2% | 211.11 | < 0.0001 |
| 94 | Mannino et al., 2008 | 1.3995 | 1.40 | [1.3035; 1.5025] | 9.27 | < 0.0001 | 58.8% | 196.80 | < 0.0001 |
| 95 | Mannino et al.2012 | 1.3820 | 1.38 | [1.2958; 1.4739] | 9.85 | < 0.0001 | 53.5% | 178.63 | < 0.0001 |
| 96 | Methvin et al., 2009 | 1.3905 | 1.39 | [1.3022; 1.4848] | 9.85 | < 0.0001 | 60.9% | 207.34 | < 0.0001 |
| 97 | Sperandio et al., 2016 | 1.3960 | 1.40 | [1.3083; 1.4896] | 10.08 | < 0.0001 | 59.5% | 207.23 | < 0.0001 |
| 98 | Triest et al., 2019 | 1.5000 | 1.50 | [1.4040; 1.6025] | 12.02 | < 0.0001 | 58.8% | 128.53 | < 0.0001 |
| 99 | Yang et al., 2020 | 1.4086 | 1.41 | [1.3196; 1.5036] | 10.29 | < 0.0001 | 54.0% | 182.71 | < 0.0001 |
| **Exclusion of studies with low quality or high risk of bias** | | | | | | | | | |
| No low quality or high risk of bias studies in this analysis | | | | | | | | | |
