# Supplementary material for: Association between hypertension and impaired lung function among adults: A systematic review and meta-analysis
Source: PLoS One. 2026 Apr 10;21(4):e0346569. doi: 10.1371/journal.pone.0346569 (PMC13068241; doi:10.1371/journal.pone.0346569)
Supplement: S3 Table — (DOCX) [file pone.0346569.s010.docx]

|  | Study Omitted | Summary OR |  | 95% CI | z | p - value | I^2^ | Q | p-value |
| --- | --- | --- | --- | --- | --- | --- | --- | --- | --- |
|  | Nothing Omitted | 2.9960 | 3.00 | [1.8637; 4.8161] | 4.53 | < 0.0001 | 84.7% | 84.83 | < 0.0001 |
| 1 | Birhan & Abebe, 2018 | 2.5845 | 2.58 | [1.7080; 3.9107] | 4.49 | < 0.0001 | 84.4% | 77.00 | < 0.0001 |
| 2 | Femi-Adeoye et al., 2024 | 3.0964 | 3.10 | [1.8244; 5.2552] | 4.19 | < 0.0001 | 85.7% | 83.68 | < 0.0001 |
| 3 | Femi-Adeoye et al., 2024 | 3.3194 | 3.32 | [1.9402; 5.6791] | 4.38 | < 0.0001 | 85.8% | 84.78 | < 0.0001 |
| 4 | Femi-Adeoye et al., 2024 | 3.3017 | 3.30 | [1.9092; 5.7100] | 4.27 | < 0.0001 | 85.8% | 84.73 | < 0.0001 |
| 5 | Ferrari et al., 2019 | 2.9468 | 2.95 | [2.0914; 4.1521] | 6.18 | < 0.0001 | 64.8% | 34.08 | 0.0007 |
| 6 | Kim et al., 2021 | 3.2567 | 3.26 | [1.8654; 5.6856] | 4.15 | < 0.0001 | 85.5% | 82.97 | < 0.0001 |
| 7 | Lee et al., 2020 | 3.3123 | 3.31 | [1.9067; 5.7539] | 4.25 | < 0.0001 | 85.8% | 84.50 | < 0.0001 |
| 8 | Patil et al., 2012 | 2.7970 | 2.80 | [1.7483; 4.4748] | 4.29 | < 0.0001 | 85.2% | 80.93 | < 0.0001 |
| 9 | Taneda et al., 2004 | 3.0144 | 3.01 | [1.7664; 5.1440] | 4.05 | < 0.0001 | 84.0% | 74.96 | < 0.0001 |
| 10 | Taneda et al., 2004 | 3.1530 | 3.15 | [1.8083; 5.4979] | 4.05 | < 0.0001 | 83.6% | 73.34 | < 0.0001 |
| 11 | Utsugi et al., 2016 | 3.3404 | 3.34 | [1.9440; 5.7399] | 4.37 | < 0.0001 | 85.8% | 84.76 | < 0.0001 |
| 12 | Yadav et al., 2015 | 2.5144 | 2.51 | [1.6954; 3.7290] | 4.59 | < 0.0001 | 83.6% | 73.12 | < 0.0001 |
| 13 | Yadav et al., 2015 | 2.9366 | 2.94 | [1.8060; 4.7750] | 4.34 | < 0.0001 | 85.6% | 83.56 | < 0.0001 |
| 14 | Yadav et al., 2015 | 2.7462 | 2.75 | [1.7541; 4.2993] | 4.42 | < 0.0001 | 85.1% | 80.56 | < 0.0001 |
| **Removing the whole study at once** | | | | | | | | | |
| 15 | Birhan & Abebe, 2018 | 2.5845 | 2.58 | [1.7080; 3.9107] | 4.49 | < 0.0001 | 84.4% | 77.00 | < 0.0001 |
| 16 | Femi-Adeoye et al., 2024 | 3.9599 | 3.96 | [1.9584; 8.0071] | 3.83 | 0.0001 | 88.0% | 83.53 | < 0.0001 |
| 17 | Ferrari et al., 2019 | 2.9468 | 2.95 | [2.0914; 4.1521] | 6.18 | < 0.0001 | 64.8% | 34.08 | 0.0007 |
| 18 | Kim et al., 2021 | 3.2567 | 3.26 | [1.8654; 5.6856] | 4.15 | < 0.0001 | 85.5% | 82.97 | < 0.0001 |
| 19 | Lee et al., 2020 | 3.3123 | 3.31 | [1.9067; 5.7539] | 4.25 | < 0.0001 | 85.8% | 84.50 | < 0.0001 |
| 20 | Patil et al., 2012 | 2.7970 | 2.80 | [1.7483; 4.4748] | 4.29 | < 0.0001 | 85.2% | 80.93 | < 0.0001 |
| 21 | Taneda et al., 2004 | 3.2281 | 3.23 | [1.7042; 6.1147] | 3.60 | 0.0003 | 81.9% | 60.89 | < 0.0001 |
| 22 | Utsugi et al., 2016 | 3.3404 | 3.34 | [1.9440; 5.7399] | 4.37 | < 0.0001 | 85.8% | 84.76 | < 0.0001 |
| 23 | Yadav et al., 2015 | 2.3118 | 2.31 | [1.5752; 3.3930] | 4.28 | < 0.0001 | 85.2% | 67.49 | < 0.0001 |
| **Exclusion of studies with low quality or high risk of bias** | | | | | | | | | |
| 25 | Birhan & Abebe, 2018 | 2.5845 | 2.58 | [1.7080; 3.9107] | 4.49 | < 0.0001 | 84.4% | 77.00 | < 0.0001 |

**S3 Table. Results of the sensitivity analysis - HT (Exposure) and ILF (Outcome) – Unadjusted analysis**
