# Supplementary material for: Association between hypertension and impaired lung function among adults: A systematic review and meta-analysis
Source: PLoS One. 2026 Apr 10;21(4):e0346569. doi: 10.1371/journal.pone.0346569 (PMC13068241; doi:10.1371/journal.pone.0346569)
Supplement: S4 Table — (DOCX) [file pone.0346569.s011.docx]

**S4 Table. Summary of subgroup analyses for the adjusted ORs (Exposure: Impaired lung function; Outcome: Hypertension)** **to explore the source of heterogeneity**

| **Factor/ Variable** | **Random Effects Model** | | | **Heterogeneity** | | | **Test for subgroup differences** | | | |
| --- | --- | --- | --- | --- | --- | --- | --- | --- | --- | --- |
|  | **Adjusted OR** | **95% CI LL** | **95% CI UL** | **I^2^ (%)** | $\boldsymbol{\tau}$**^2^** | **p** | **χ^2^** | **df** | **p** |  |
|  |  |  |  |  |  |  |  |  |  |  |
| **Study Design** | 1.3986 | 1.3107 | 1.4925 | 59.8 | 0.0362 | < 0.0001 | 3.09 | 2 | 0.2128 |  |
| Case-Control Study (k = 1) | 3.6600 | 1.2477 | 10.7361 | -- | -- |  |  |  |  |  |
| Cross-sectional Study (k = 81) | 1.3944 | 1.2989 | 1.4969 | 58.7 | 0.0410 | < 0.0001 |  |  |  |  |
| Cohort Study (k = 4) | 1.3838 | 1.1875 | 1.6125 | 79.5 | 0.0196 | 0.0022 |  |  |  |  |
|  |  |  |  |  |  |  |  |  |  |  |
| **Country** | 1.3986 | 1.3107 | 1.4925 | 59.8 | 0.0362 | < 0.0001 | 42.36 | 32 | 0.1041 |  |
| Italy (k = 1) | 3.6600 | 1.2477 | 10.7361 | -- | -- |  |  |  |  |  |
| United States (k = 14) | 1.4084 | 1.2252 | 1.6188 | 72.8 | 0.0317 | < 0.0001 |  |  |  |  |
| Korea (k = 2) | 1.7793 | 1.5326 | 2.0658 | 0 | 0 | 0.6318 |  |  |  |  |
| Iran (k = 1) | 1.4300 | 1.0238 | 1.9974 | -- | -- |  |  |  |  |  |
| Turkey (k = 2) | 1.2665 | 0.5163 | 3.1069 | 80.1 | 0.3380 | 0.0251 |  |  |  |  |
| Algeria (k = 2) | 1.7851 | 1.0327 | 3.0858 | 56.1 | 0.0910 | 0.1314 |  |  |  |  |
| Norway (k = 2) | 1.0113 | 0.6671 | 1.5332 | 0 | 0 | 0.9085 |  |  |  |  |
| Malawi (k = 2) | 0.8668 | 0.1630 | 4.6094 | 82.3 | 1.2145 | 0.0176 |  |  |  |  |
| South Africa (k = 2) | 0.9896 | 0.6134 | 1.5965 | 70.2 | 0.0841 | 0.0671 |  |  |  |  |
| Krygyztan (k = 4) | 1.5015 | 1.1545 | 1.9529 | 18.8 | 0.0052 | 0.2963 |  |  |  |  |
| Sri Lanka (k = 2) | 1.0961 | 0.7315 | 1.6424 | 0 | 0 | 0.7592 |  |  |  |  |
| Benin (k = 2) | 1.1283 | 0.7604 | 1.6743 | 13.7 | 0.0122 | 0.2817 |  |  |  |  |
| Morocco (k = 2) | 1.2796 | 0.8657 | 1.8914 | 26.8 | 0.0214 | 0.2423 |  |  |  |  |
| China (k = 2) | 1.6364 | 1.0157 | 2.6364 | 0 | 0 | 0.5549 |  |  |  |  |
| Germany (k = 2) | 1.3594 | 0.8647 | 2.1371 | 0 | 0 | 0.6113 |  |  |  |  |
| Poland (k = 2) | 1.1870 | 0.4726 | 2.9813 | 76.5 | 0.3380 | 0.0391 |  |  |  |  |
| Portugal (k = 2) | 1.3658 | 0.7781 | 2.3972 | 75 | 0.1287 | 0.0454 |  |  |  |  |
| United Kingdom (k = 2) | 1.0540 | 0.4182 | 2.6565 | 85.9 | 0.3826 | 0.0077 |  |  |  |  |
| Netherland (k = 2) | 1.7156 | 1.1487 | 2.5622 | 0 | 0 | 0.7552 |  |  |  |  |
| Philippines (k = 4) | 1.4118 | 1.1157 | 1.7866 | 35.5 | 0.0054 | 0.1989 |  |  |  |  |
| India (k = 6) | 1.4332 | 1.1325 | 1.8137 | 41.9 | 0.0211 | 0.1261 |  |  |  |  |
| Malaysia (k = 2) | 1.2001 | 0.7308 | 1.9706 | 20.1 | 0.0381 | 0.2633 |  |  |  |  |
| Iceland (k = 2) | 1.8007 | 1.0779 | 3.0085 | 46.9 | 0.0644 | 0.1698 |  |  |  |  |
| Saudi Arabia (k = 2) | 1.1583 | 0.8077 | 1.6609 | 0 | 0 | 0.7151 |  |  |  |  |
| Austria (k = 2) | 1.0461 | 0.7878 | 1.3892 | 0 | 0 | 0.4095 |  |  |  |  |
| Tunisia (k = 2) | 1.9482 | 1.3184 | 2.8788 | 0 | 0 | 0.3617 |  |  |  |  |
| Australia (k = 2) | 0.8173 | 0.3774 | 1.7698 | 52.2 | 0.1636 | 0.1481 |  |  |  |  |
| Estonia (k = 2) | 1.1129 | 0.5583 | 2.2186 | 45.3 | 0.1129 | 0.1764 |  |  |  |  |
| Albania (k = 2) | 2.0991 | 1.0972 | 4.0161 | 86.5 | 0.1904 | 0.0065 |  |  |  |  |
| Sweden (k = 5) | 1.3484 | 1.1799 | 1.5409 | 0 | 0 | 0.9047 |  |  |  |  |
| Canada (k = 2) | 1.2684 | 0.8260 | 1.9475 | 0 | 0 | 0.9717 |  |  |  |  |
| Selected Countries (k = 2) | 1.7294 | 1.2061 | 2.4797 | 92.2 | 0.0623 | 0.0003 |  |  |  |  |
| Brazil (k = 1) | 17.5130 | 1.6592 | 184.82 | -- | -- |  |  |  |  |  |
|  |  |  |  |  |  |  |  |  |  |  |
| **Lung Function Impairment Type** | 1.3986 | 1.3107 | 1.4925 | 59.8 | 0.0362 | < 0.0001 | 10.02 | 1 | 0.0015 |  |
| Obstructive (k = 49) | 1.2758 | 1.1584 | 1.4052 | 61.6 | 0.0527 | < 0.0001 |  |  |  |  |
| Restrictive (k = 37) | 1.5632 | 1.4422 | 1.6944 | 30.3 | 0.0137 | 0.0439 |  |  |  |  |
|  |  |  |  |  |  |  |  |  |  |  |
| **Adjusted for Alcohol Consumption Levels** | 1.3986 | 1.3107 | 1.4925 | 59.8 | 0.0362 | < 0.0001 | 18.87 | 1 | < 0.0001 |  |
| Yes (k = 1) | 1.1100 | 1.0191 | 1.2090 | -- | -- |  |  |  |  |  |
| No (k = 85) | 1.4086 | 1.3196 | 1.5036 | 54.0 | 0.0349 | < 0.0001 |  |  |  |  |
|  |  |  |  |  |  |  |  |  |  |  |
| **Adjusted for Sex** | 1.3986 | 1.3107 | 1.4925 | 59.8 | 0.0362 | < 0.0001 | 7.49 | 1 | 0.0062 |  |
| Yes (k = 82) | 1.3630 | 1.2780 | 1.4538 | 50.6 | 0.0282 | < 0.0001 |  |  |  |  |
| No (k = 4) | 1.7488 | 1.4806 | 2.0656 | 77.3 | 0.0207 | 0.0042 |  |  |  |  |
|  |  |  |  |  |  |  |  |  |  |  |
| **Adjusted for Education** | 1.3986 | 1.3107 | 1.4925 | 59.8 | 0.0362 | < 0.0001 | 5.07 | 1 | 0.0244 |  |
| Yes (k = 75) | 1.3547 | 1.2622 | 1.4540 | 52.4 | 0.0338 | < 0.0001 |  |  |  |  |
| No (k = 11) | 1.6087 | 1.4100 | 1.8355 | 64.9 | 0.0240 | 0.0015 |  |  |  |  |
|  |  |  |  |  |  |  |  |  |  |  |
| **Adjusted for Macro nutrient intake: Protein, carbohydrate, total fat** | 1.3986 | 1.3107 | 1.4925 | 59.8 | 0.0362 | < 0.0001 | 18.87 | 1 | < 0.0001 |  |
| Yes (k =1) | 1.1100 | 1.0191 | 1.2090 | -- | -- |  |  |  |  |  |
| No (k = 85) | 1.4086 | 1.3196 | 1.5036 | 54.0 | 0.0349 | < 0.0001 |  |  |  |  |
|  |  |  |  |  |  |  |  |  |  |  |
| **Adjusted for House hold Income level** | 1.3986 | 1.3107 | 1.4925 | 59.8 | 0.0362 | < 0.0001 | 18.87 | 1 | < 0.0001 |  |
| Yes (k =1) | 1.1100 | 1.0191 | 1.2090 | -- | -- |  |  |  |  |  |
| No (k = 85) | 1.4086 | 1.3196 | 1.5036 | 54.0 | 0.0349 | < 0.0001 |  |  |  |  |
|  |  |  |  |  |  |  |  |  |  |  |
| **Adjusted for physical inactivity** | 1.3986 | 1.3107 | 1.4925 | 59.8 | 0.0362 | < 0.0001 | 4.42 | 1 | 0.0355 |  |
| Yes (k = 1) | 17.5130 | 1.6592 | 184.8463 | -- | -- |  |  |  |  |  |
| No (k = 85) | 1.3960 | 1.3083 | 1.4896 | 59.5 | 0.0361 | < 0.0001 |  |  |  |  |
|  |  |  |  |  |  |  |  |  |  |  |
| **Adjusted for Obesity** | 1.3986 | 1.3107 | 1.4925 | 59.8 | 0.0362 | < 0.0001 | 4.42 | 1 | 0.0355 |  |
| Yes (k = 1) | 17.5130 | 1.6592 | 184.8463 | -- | -- |  |  |  |  |  |
| No (k = 85) | 1.3960 | 1.3083 | 1.4896 | 59.5 | 0.0361 | < 0.0001 |  |  |  |  |
|  |  |  |  |  |  |  |  |  |  |  |
| **Adjusted for BMI** | 1.3986 | 1.3107 | 1.4925 | 59.8 | 0.0362 | < 0.0001 | 1.02 | 1 | 0.3130 |  |
| Yes (k = 84) | 1.3869 | 1.2996 | 1.4802 | 58.3 | 0.0348 | < 0.0001 |  |  |  |  |
| No (k = 2) | 4.1285 | 0.4965 | 34.3278 | 71.3 | 1.8102 | 0.0619 |  |  |  |  |
|  |  |  |  |  |  |  |  |  |  |  |
| **Adjusted for Diabetes Status** | 1.3986 | 1.3107 | 1.4925 | 59.8 | 0.0362 | < 0.0001 | 1.01 | 1 | 0.3138 |  |
| Yes (k = 4) | 2.0138 | 0.9886 | 4.1022 | 86.4 | 0.3557 | < 0.0001 |  |  |  |  |
| No (k = 82) | 1.3948 | 1.3055 | 1.4902 | 53.1 | 0.0349 | < 0.0001 |  |  |  |  |
|  |  |  |  |  |  |  |  |  |  |  |
| **Adjusted for Smoking** | 1.3986 | 1.3107 | 1.4925 | 59.8 | 0.0362 | < 0.0001 | 2.32 | 1 | 0.1275 |  |
| Yes (k = 84) | 1.3852 | 1.2980 | 1.4782 | 58.6 | 0.0346 | < 0.0001 |  |  |  |  |
| No (k = 2) | 2.1006 | 1.2345 | 3.5740 | 34.0 | 0.0804 | 0.2184 |  |  |  |  |
|  |  |  |  |  |  |  |  |  |  |  |
| **Adjusted for Race** | 1.3986 | 1.3107 | 1.4925 | 59.8 | 0.0362 | < 0.0001 | 0.40 | 1 | 0.5265 |  |
| Yes (k = 6) | 1.3332 | 1.1473 | 1.5491 | 86.8 | 0.0253 | < 0.0001 |  |  |  |  |
| No (k = 80) | 1.4068 | 1.3096 | 1.5113 | 51.5 | 0.0402 | < 0.0001 |  |  |  |  |
|  |  |  |  |  |  |  |  |  |  |  |
| **Adjusted for Dyslipidemia** | 1.3986 | 1.3107 | 1.4925 | 59.8 | 0.0362 | < 0.0001 | 0.42 | 1 | 0.5153 |  |
| Yes (k =2) | 3.3939 | 0.2388 | 48.2418 | 81.0 | 3.0811 | 0.0219 |  |  |  |  |
| No (k = 84) | 1.4060 | 1.3172 | 1.5007 | 53.5 | 0.0347 | < 0.0001 |  |  |  |  |
