# Supplementary material for: Association between hypertension and impaired lung function among adults: A systematic review and meta-analysis
Source: PLoS One. 2026 Apr 10;21(4):e0346569. doi: 10.1371/journal.pone.0346569 (PMC13068241; doi:10.1371/journal.pone.0346569)
Supplement: S5 Table — (DOCX) [file pone.0346569.s012.docx]

**S5 Table. Summary of subgroup analyses for the adjusted ORs (Exposure: Hypertension; Outcome: Impaired lung function) to explore the source of heterogeneity**

| **Factor/ Variable** | **Random Effects Model** | | | **Heterogeneity** | | | **Test for subgroup differences** | | |
| --- | --- | --- | --- | --- | --- | --- | --- | --- | --- |
|  | **Adjusted OR** | **95% CI LL** | **95% CI UL** | **I^2^ (%)** | $\boldsymbol{\tau}$**^2^** | **p** | **χ^2^** | **df** | **p** |
|  |  |  |  |  |  |  |  |  |  |
| **Study Design** | 1.9417 | 1.5110 | 2.4953 | 0.0 | 0 | 0.5992 | 0.00 | 0 | -- |
| Cross-sectional Study (k = 5) | 1.9417 | 1.5110 | 2.4953 |  | 0 |  |  |  |  |
|  |  |  |  |  |  |  |  |  |  |
| **Country** | 1.9417 | 1.5110 | 2.4953 | 0.0 | 0 | 0.5992 | 2.76 | 4 | 0.5992 |
| Nigeria (k = 1) | 2.9950 | 1.2469 | 7.1937 | -- | -- |  |  |  |  |
| Korea (k = 2) | 1.6576 | 1.1384 | 2.4135 | 0.0 | 0 | 0.3533 |  |  |  |
| United States (k = 2) | 2.0911 | 1.4517 | 3.0121 | 0.0 | 0 | 0.7326 |  |  |  |
| Japan (k = 0) | NA |  |  | -- | -- |  |  |  |  |
|  |  |  |  |  |  |  |  |  |  |
| **Lung Function Impairment Type** | 1.9417 | 1.5110 | 2.4953 | 0.0 | 0 | 0.5992 | 1.25 | 2 | 0.5358 |
| Obstructive (k = 2) | 2.0331 | 1.4145 | 2.9223 | 0.0 | 0 | 0.9009 |  |  |  |
| Restrictive (k = 2) | 1.7292 | 1.1182 | 2.6740 | 22.0 | 0.0230 | 0.2576 |  |  |  |
| Restrictive and Obstructive (k = 1) | 2.9950 | 1.2469 | 7.1937 | -- | -- |  |  |  |  |
|  |  |  |  |  |  |  |  |  |  |
| **Continent** | 1.9417 | 1.5110 | 2.4953 | 0.0 | 0 | 0.5992 | 1.78 | 2 | 0.4108 |
| Africa (k = 1) | 2.9950 | 1.2469 | 7.1937 | -- | -- |  |  |  |  |
| Asia (k = 2) | 1.6576 | 1.1384 | 2.4135 | 0.0 | 0 | 0.3533 |  |  |  |
| North America (k = 2) | 2.0911 | 1.4517 | 3.0121 | 0.0 | 0 | 0.7326 |  |  |  |
|  |  |  |  |  |  |  |  |  |  |
| **Adjusted for Sex** | 1.9417 | 1.5110 | 2.4953 | 0.0 | 0 | 0.5992 | 1.02 | 1 | 0.3117 |
| No (k = 1) | 2.9950 | 1.2469 | 7.1937 | -- | -- |  |  |  |  |
| Yes (k = 4) | 1.8681 | 1.4378 | 2.4270 | 0.0 | 0 | 0.6294 |  |  |  |
|  |  |  |  |  |  |  |  |  |  |
| **Adjusted for BMI** | 1.9417 | 1.5110 | 2.4953 | 0.0 | 0 | 0.5992 | 0.00 | 0 | -- |
| Yes (k = 5) | 1.9417 | 1.5110 | 2.4953 | 0.0 | 0 | 0.5992 |  |  |  |
| No (k = 0) | NA |  |  |  |  |  |  |  |  |
|  |  |  |  |  |  |  |  |  |  |
| **Adjusted for Education** | 1.9417 | 1.5110 | 2.4953 | 0.0 | 0 | 0.5992 | 0.07 | 1 | 0.7885 |
| No (k = 4) | 1.9127 | 1.4546 | 2.5152 | 0.0 | 0 | 0.4427 |  |  |  |
| Yes (k = 1) | 2.1000 | 1.1239 | 3.9237 | -- | -- |  |  |  |  |
|  |  |  |  |  |  |  |  |  |  |
| **Adjusted for Physical Inactivity** | 1.9417 | 1.5110 | 2.4953 | 0.0 | 0 | 0.5992 | 2.07 | 1 | 0.1500 |
| No (k = 4) | 2.1810 | 1.6213 | 2.9340 | 0.0 | 0 | 0.8767 |  |  |  |
| Yes (k = 1) | 1.4500 | 0.9062 | 2.3202 | -- | -- |  |  |  |  |
|  |  |  |  |  |  |  |  |  |  |
| **Adjusted for Alcohol Consumption** | 1.9417 | 1.5110 | 2.4953 | 0.0 | 0 | 0.5992 | 1.02 | 1 | 0.3117 |
| No (k = 1) | 2.9950 | 1.2469 | 7.1937 | -- | -- |  |  |  |  |
| Yes (k = 4) | 1.8681 | 1.4378 | 2.4270 | 0.0 | 0 | 0.6294 |  |  |  |
|  |  |  |  |  |  |  |  |  |  |
| **Adjusted for Diabetes Status** | 1.9417 | 1.5110 | 2.4953 | 0.0 | 0 | 0.5992 | 0.19 | 1 | 0.6649 |
| No (k = 3) | 1.8585 | 1.2593 | 2.7429 | 14.5 | 0.0212 | 0.3103 |  |  |  |
| Yes (k = 2) | 2.0911 | 1.4517 | 3.0121 | 0.0 | 0 | 0.7326 |  |  |  |
|  |  |  |  |  |  |  |  |  |  |
| **Adjusted for dyslipidemia** | 1.9417 | 1.5110 | 2.4953 | 0.0 | 0 | 0.5992 | 0.00 | 0 | -- |
| No (k = 5) | 1.9417 | 1.5110 | 2.4953 | 0.0 | 0 | 0.5992 |  |  |  |
| Yes (k = 0) | NA |  |  | -- | -- |  |  |  |  |
|  |  |  |  |  |  |  |  |  |  |
| **Adjusted for Income** | 1.9417 | 1.5110 | 2.4953 | 0.0 | 0 | 0.5992 | 0.07 | 1 | 0.7885 |
| No (k = 4) | 1.9127 | 1.4546 | 2.5152 | 0.0 | 0 | 0.4427 |  |  |  |
| Yes (k = 1) | 2.1000 | 1.1239 | 3.9237 | -- | -- |  |  |  |  |
|  |  |  |  |  |  |  |  |  |  |
| **Adjusted for Residence** | 1.9417 | 1.5110 | 2.4953 | 0.0 | 0 | 0.5992 | 0.07 | 1 | 0.7885 |
| No (k = 4) | 1.9127 | 1.4546 | 2.5152 | 0.0 | 0 | 0.4427 |  |  |  |
| Yes (k = 1) | 2.1000 | 1.1239 | 3.9237 | -- | -- |  |  |  |  |
|  |  |  |  |  |  |  |  |  |  |
| **Adjusted for Waist Circumference** | 1.9417 | 1.5110 | 2.4953 | 0.0 | 0 | 0.5992 | 0.07 | 1 | 0.7895 |
| Yes (k = 2) | 1.8892 | 0.9529 | 3.7452 | 51.1 | 0.1344 | 0.1528 |  |  |  |
| No (k = 3) | 2.0934 | 1.5275 | 2.8689 | 0.0 | 0 | 0.9432 |  |  |  |
|  |  |  |  |  |  |  |  |  |  |
| **Adjusted for Smoking** | 1.9417 | 1.5110 | 2.4953 | 0.0 | 0 | 0.5992 | 1.02 | 1 | 0.3117 |
| No (k = 1) | 2.9950 | 1.2469 | 7.1937 | -- | -- |  |  |  |  |
| Yes (k = 4) | 1.8681 | 1.4378 | 2.4270 | 0.0 | 0 | 0.6294 |  |  |  |
|  |  |  |  |  |  |  |  |  |  |
| **Adjusted for Race** | 1.9417 | 1.5110 | 2.4953 | 0.0 | 0 | 0.5992 | 0.00 | 1 | -- |
| No (k = 5) | 1.9417 | 1.5110 | 2.4953 | 0.0 | 0 | 0.5992 |  |  |  |
|  |  |  |  |  |  |  |  |  |  |
| **Adjusted for Obesity** | 1.9417 | 1.5110 | 2.4953 | 0.0 | 0 | 0.5992 | 0.00 | 1 | -- |
| No (k = 5) | 1.9417 | 1.5110 | 2.4953 | 0.0 | 0 | 0.5992 |  |  |  |
|  |  |  |  |  |  |  |  |  |  |
| **Adjusted for Macro Nutrient Intake** | 1.9417 | 1.5110 | 2.4953 | 0.0 | 0 | 0.5992 | 0.00 | 1 | -- |
| No (k = 5) | 1.9417 | 1.5110 | 2.4953 | 0.0 | 0 | 0.5992 |  |  |  |
