# Supplementary material for: Association between hypertension and impaired lung function among adults: A systematic review and meta-analysis
Source: PLoS One. 2026 Apr 10;21(4):e0346569. doi: 10.1371/journal.pone.0346569 (PMC13068241; doi:10.1371/journal.pone.0346569)
Supplement: Attachment — Submitted filename: Response to Reviewers.docx [file pone.0346569.s014.docx]

M.L. Dilakshi Lekamge
Department of Public Health,
Faculty of Medicine,
University of Kelaniya,
Sri Lanka.
[dilakshi.lekamge@gmail.com](mailto:dilakshi.lekamge@gmail.com)
15^th^ January 2026

The Editor in Chief,
Editorial Office,
PLOS One.

Dear Editor,

**Subject: Response to Reviewers’ Comments on the manuscript titled “Association between hypertension and impaired lung function among adults: A systematic review and meta-analysis” – Manuscript ID: PONE-D-25-57756**

We would like to express our sincere gratitude to the Editorial Office and the reviewers for their careful evaluation and constructive feedback on our manuscript. We are grateful that the reviewer considered our systematic literature review and meta-analysis to be comprehensive and acknowledged our efforts to explore the bidirectional association between impaired lung function and hypertension.

We have carefully revised the manuscript in response to the reviewer’s comments. A detailed, point by point response to each comment from the reviewer 1 is provided below. All corresponding revisions have been incorporated into the revised manuscript and are clearly indicated. We believe these changes have strengthened the clarity, methodological rigor, and overall quality of the paper.

**Reviewer comment 1:** The meta-analysis and review of literature is good and highlights the efforts taken by the authors to show the bidirectional association between impaired lung functions and hypertension.

**Response for comment 1:** We sincerely thank the reviewer for the positive feedback.

**Reviewer comment 2:** There is too much of reliance on heterogeneous observational studies and limits causal inference.

**Response for comment 2:** We fully agree with the reviewer that the observational nature of the included studies limits causal inference as our meta-analysis is primarily based on observational studies. Although the inclusion of a single cohort study allowed some assessment of temporal relationship, residual confounding cannot be excluded, and definitive causality can not be established. We have clarified this point in the abstract and the discussion of the revised manuscript and emphasized that our findings indicate associations rather than causal relationships.

The conclusion section of the abstract has been updated with the following text: “This study concludes that there is a significant positive relationship between impaired lung function and hypertension, with temporal evidence suggesting that impaired lung function may precede hypertension, while the reverse causal direction lacks such evidence.”

The discussion section was updated with the following text: “All of the included studies were observational in design; therefore, causal inference is limited. Although inclusion of a single cohort study provided evidence of a temporal relationship, residual and unmeasured confounding cannot be excluded and observed associations should not be interpreted as definitive evidence of causality.”

**Reviewer comment 3:** There would have been lot of differences in comorbidities like smoking burden, obesity, underlying respiratory conditions, metabolic syndrome which may not have been accounted for.

**Response for comment 3:** We agree that these comorbidities may influence both lung function and hypertension. Unfortunately, these variables were not uniformly reported across studies, limiting our ability to perform adjusted pooled analyses. However, wherever available, we extracted multivariable-adjusted estimates that account for major confounders. We have now clearly acknowledged in the discussion that residual confounding from such comorbidities remains possible.

The discussion section was revised with the following text: **“**Important comorbidities such as smoking burden, obesity, underlying respiratory conditions and metabolic syndrome were not uniformly reported across studies. As a result, comprehensive adjustment for these factors was not possible, and residual confounding cannot be excluded.”

**Reviewer comment 4:** The authors are also not clear in their definition about impaired lung functions.

**Response for comment 4:** We thank the reviewer for this comment. We agree that our original description of impaired lung function lacked clarity and we have now revised it under the selection criteria of the methods section as follows: “We only accepted the articles where, impaired lung function was defined according to the criteria used in the individual studies, provided that impairment was based on FEV_1_ (forced expiratory volume in 1 second), FVC (forced vital capacity) and FEV_1_/FVC spirometric measurements. Studies were included if they reported impaired lung function as obstructive, restrictive, or mixed pattern, or if impairment was defined using accepted thresholds such as FEV_1_/FVC < 0.70 or values below the lower limit of normal (LLN, <5^th^ percentile) of the FEV_1_/FVC ratio calculated from the reference equations.”

**Reviewer comment 5:** The spirometers were not standardised, and this may affect pooled estimates.

**Response for comment 5:** We agree that lack of standardization of spirometry equipment and calibration across studies may have introduced variability in lung function measurements. Details regarding device type and calibration procedures were not consistently reported. Therefore, that limited our ability to assess this source of bias quantitatively. These methodological variations may have contributed to heterogeneity and could have influenced the pooled effect estimates. We have now added this clarification to the limitations paragraph of the strengths and limitations under the discussion.

**Reviewer comment 6:** Important confounders like level of physical activity, environmental or occupational exposure, effect of medications were not taken into account.

**Response for comment 6:** We agree with this important point. These variables were inconsistently reported across studies and therefore could not be comprehensively included in subgroup or meta regression analyses. We have now added this under limitations acknowledging the potential influence of unmeasured or residual confounding related to physical activity, occupational or environmental exposures, and effect of medication.

**Reviewer comment 7:** Despite trying to retrieve a lot of raw data, null or negative studies may be underestimated.

**Response for comment 7:** We agree with the reviewer that studies with null or negative findings may be under-represented in the available literature, despite our efforts to obtain unpublished data from authors. This under representation may contribute to publication bias and could potentially lead to an overestimation of the pooled effect size. To minimize this risk, we conducted comprehensive database searches, reviewed reference lists, and attempted to retrieve unpublished data where possible. We have now strengthened the limitations section to reflect that the under-representation of null or negative studies cannot be fully excluded.

We believe that the revised manuscript has appropriately addressed all comments raised by the reviewers. We are grateful for the opportunity to revise and resubmit our manuscript with the revisions.

Thank you for your continued consideration of our manuscript.

Yours sincerely,

M.L. Dilakshi Lekamge

[On behalf of all co-authors]
