# Supplementary material for: Association between hypertension and impaired lung function among adults: A systematic review and meta-analysis
Source: PLoS One. 2026 Apr 10;21(4):e0346569. doi: 10.1371/journal.pone.0346569 (PMC13068241; doi:10.1371/journal.pone.0346569)
Supplement: Attachment — Submitted filename: Response_to_Reviewers_auresp_2.docx [file pone.0346569.s015.docx]

M.L. Dilakshi Lekamge
Department of Public Health,
Faculty of Medicine,
University of Kelaniya,
Sri Lanka.
[dilakshi.lekamge@gmail.com](mailto:dilakshi.lekamge@gmail.com)
07^th^ March 2026

The Editor in Chief,
Editorial Office,
PLOS One.

Dear Editor,

**Subject: Response to Reviewers’ Comments on the manuscript titled “Association between hypertension and impaired lung function among adults: A systematic review and meta-analysis” – Manuscript ID: PONE-D-25-57756R1**

We would like to express our sincere gratitude to the Editorial Office and both reviewers for their careful evaluation and constructive feedback on our manuscript. We are particularly thankful for reviewer 1 for confirming that all previous comments have been satisfactorily addressed. We also greatly appreciate reviewer 2’s positive assessment of our systematic review and meta-analysis as a comprehensive and clinically relevant contribution to cardiovascular-respiratory epidemiology examining the association between impaired lung function and hypertension. We are grateful for the reviewer’s insightful suggestions, which have helped us further improve the clarity, interpretation, and overall quality of the manuscript.

We have carefully revised the manuscript in response to all comments raised in this review round. A detailed, point by point response to each comment from both reviewers are provided below. All corresponding revisions have been incorporated into the revised manuscript and are clearly indicated using track changes. We believe these revisions have strengthened the clarity, methodological rigor, and overall quality of the paper.

**Reviewer 1 overall comment:** All comments have been addressed to satisfaction, and the manuscript may be accepted if found satisfactory by other members of the reviewing committee and the editorial board.

**Response for Reviewer 1 overall comment:** We sincerely thank the reviewer for the positive feedback on our manuscript and for confirming that all previous comments have been satisfactorily addressed.

**Reviewer 2 overall comment:** This manuscript presents a comprehensive and timely systematic review and meta-analysis examining the bidirectional association between impaired lung function (ILF) and hypertension (HTN). The work is ambitious in scope, methodologically structured, and clinically relevant. The inclusion of both directions of association and the attempt to explore heterogeneity through subgroup and meta-regression analyses are notable strengths. The manuscript makes a meaningful contribution to cardiovascular–respiratory epidemiology. However, several methodological clarifications, interpretative refinements, and structural improvements are required before publication.

**Response for Reviewer 2 comment:** We thank the reviewer for the thoughtful and constructive feedback. We have addressed each comment in detail below.

**Reviewer 2 comment 1: Conceptual Framing of “Bidirectional” Association**
The manuscript repeatedly refers to a “bidirectional relationship,” yet: The majority of included studies are cross-sectional. Only one cohort study examined ILF → HTN temporality. There is no longitudinal evidence supporting HTN → ILF direction. Thus, “bidirectional association” may overstate the strength of causal inference.

Recommendation: Rephrase to: “Evidence supports a significant association in both directions, with stronger temporal evidence for impaired lung function preceding hypertension.” In the Discussion and Conclusion, clearly distinguish: Association (supported), Causality (not established except limited evidence for ILF → HTN)

**Response for Reviewer 2 comment 1:** We agree with the reviewer that the term “bidirectional association” may overstate what is supported by the available evidence. We have clarified this point in the abstract, discussion, and the conclusion of the revised manuscript and emphasized that our findings indicate associations rather than causal relationships.

The conclusion section of the abstract has been revised with the following text: “This study concludes that there is a significant positive association between impaired lung function and hypertension in both directions, with stronger temporal evidence suggesting that impaired lung function may precede hypertension, while the reverse causal direction lacks such evidence.”

The discussion section was updated with the following text: “Since cross-sectional studies do not support temporal associations, the findings should be interpreted cautiously. Limited longitudinal evidence from a single cohort study suggests that impaired lung function may precede hypertension; however, comparable longitudinal evidence is lacking to support temporality in the reverse direction. Therefore, these results support significant associations in both directions but do not establish causality except limited evidence when impaired lung function is considered the exposure.”

We have revised the limitations section to clarify that causal inference is limited due to the predominance of cross-sectional studies and to emphasize that longitudinal evidence supporting temporality is limited to impaired lung function preceding hypertension. The limitation section under the discussion section has been revised with the following text: “All of the included studies were observational in design, and the majority were cross-sectional, limiting causal inference.”

The conclusion section of paper has been revised with the following text: “In conclusion, this systematic review and meta-analysis provides the first comprehensive evidence of a significant positive association between impaired lung function and hypertension in both directions. The available limited prospective evidence suggests impaired lung function contributes to subsequent hypertension risk, while support for the opposite causal direction is lacking.”

**Reviewer 2 comment 2: Clinical Interpretation of Effect Sizes**

Key Findings: ILF → HTN (adjusted OR): 1.40, HTN → ILF (adjusted OR): 1.94

Concern: The manuscript suggests hypertension has a “more substantial association” because OR is numerically higher. However: Different sample sizes, Different heterogeneity profiles, Stronger publication bias in HTN → ILF crude models, Only 6 adjusted studies in HTN → ILF analysis, Direct comparison of magnitude may be misleading.

Recommendation: Add clarification: “Although adjusted effect sizes were numerically larger when hypertension was the exposure, differences in study design, number of included studies, and publication bias limit direct comparison of magnitude between directions.”

**Response for Reviewer 2 comment 2:** We totally agree with the above concern, and we have revised the Discussion section of the paper to clarify that although adjusted effect sizes were numerically larger when hypertension was the exposure, differences in study characteristics and potential publication bias limit direct comparison of magnitude between directions. The Discussion has been revised with the following text: “Although adjusted effect sizes were numerically larger when hypertension was considered as the exposure, differences in study design, number of included studies, and publication bias limit direct comparison of magnitude between the two directions.”

**Reviewer 2 comment 3:** **High Heterogeneity (I² > 80%)**

Several analyses show substantial heterogeneity: ILF → HTN (crude): I² = 84%
HTN → ILF (crude): I² = 84.7%

Concern: Despite subgroup and meta-regression analyses, residual heterogeneity remains high.

Recommendations: Provide clearer interpretation: Clinical heterogeneity (definitions of ILF), Population differences, Variability in spirometry reference equations, Differences in
hypertension measurement protocols

Explicitly state: “The pooled estimate should be interpreted as an average association across heterogeneous populations rather than a precise universal effect size.”

**Response for Reviewer 2 comment 3:** We thank the reviewer for this valuable comment. In response, we have expanded the Discussion to provide a clearer interpretation of the residual heterogeneity observed in our analyses. Specifically, we have elaborated on potential sources of clinical, methodological, and population heterogeneity, including differences in definitions of impaired lung function, variability in spirometry reference equations, differences in population characteristics, and variations in hypertension measurement protocols.

The following text has been added to the Discussion section of the manuscript: “In addition, residual heterogeneity may reflect broader clinical and methodological diversity across studies, including differences in the definition of impaired lung function based on fixed ratio or lower limit of normal criteria, variability in spirometry reference equations, and differences in population characteristics such as age distribution, comorbidity profiles, and geographic settings. Furthermore, residual heterogeneity may have arisen from differences in hypertension measurement protocols across studies, including variations in measurement devices, the timing and setting of blood pressure assessment, the number of readings obtained, and whether hypertension status was determined through direct measurement, medication use, or self-reported physician diagnosis. These methodological variations may have contributed to heterogeneity and could have influenced the pooled effect estimates. Therefore, pooled estimate should be interpreted as an average association across heterogeneous populations rather than a precise universal effect size.”

**Reviewer 2 comment 4:** **Restrictive Lung Impairment Signal**

You consistently report that restrictive impairment shows stronger association with HTN.
This is clinically important and deserves deeper discussion.

Suggested Expansion: Discuss potential mechanisms:
Obesity-related restrictive physiology
Metabolic syndrome clustering
Low-grade systemic inflammation
Diaphragmatic mechanics
Vascular stiffness and reduced lung compliance
Currently, this finding is mentioned but not mechanistically elaborated enough.
This could become one of the manuscript’s key contributions.

**Response for Reviewer 2 comment 4:** We thank the reviewer for this valuable suggestion. We agree that the stronger association observed between restrictive lung impairment and hypertension is clinically important and warrants further mechanistic discussion. Accordingly, we have expanded the Discussion section to provide a more detailed explanation of potential biological pathways underlying this relationship. We have specifically elaborated on obesity related restrictive respiratory physiology, including reductions in lung volumes and mechanical limitations of the thoraco-abdominal system, the role of metabolic syndrome clustering, the contribution of low-grade systemic inflammation driven by adipose tissue dysfunction, the impact of altered diaphragmatic mechanics and the effects of vascular stiffness and reduced lung compliance.

These additions provide biological plausibility for our findings and highlight the potential role of restrictive lung impairment as a clinical marker of systemic cardiometabolic dysfunction and provide a descriptive explanation on why restrictive impairment may demonstrate a more consistent and robust association with hypertension compared to obstructive impairment.

The revised text has been added to the Discussion section of the revised manuscript with track changes (Page 40 and 41, Lines 850 – 889). We have also highlighted this observation as one of the key contributions of the present study.

**Reviewer 2 comment 5:** **Publication Bias Interpretation**

For HTN → ILF (crude): Egger’s test significant, Trim-and-fill changes significance, Yet you conclude: “Adjustment does not change overall conclusion.” This is inaccurate because: After trim-and-fill, p = 0.280 (non-significant)

Recommendation: Revise to: “After adjusting for potential publication bias, the crude association between hypertension and impaired lung function lost statistical significance, suggesting that small-study effects may have inflated the initial estimate.”
This is important for credibility.

**Response for Reviewer 2 comment 5:** We thank the reviewer for this important observation. We agree that our original wording did not accurately reflect the impact of potential publication bias on the crude association between hypertension and impaired lung function. Accordingly, we have now revised the relevant section of the manuscript to clarify that the Egger’s test indicated possible small-study effects and after applying the trim-and-fill method, the pooled crude association was no longer statistically significant.

The revised section is as follows: “After adjusting for potential publication bias, the crude association between hypertension and impaired lung function lost statistical significance, suggesting that small-study effects may have inflated the initial estimate.”

**Reviewer 2 comment 6:** **Meta-Regression Interpretation**

Your multivariable meta-regression:
Explained 32.56% heterogeneity
No moderators statistically significant
Clarification Needed

The manuscript suggests sex and education reduce effect size in univariate models but then are not significant in multivariable analysis. Explain: Collinearity, Reduced power, Limited study-level variability. Otherwise, interpretation appears inconsistent.

**Response for Reviewer 2 comment 6:** We agree with the reviewer that clarification is needed regarding the interpretation of univariate and multivariable meta-regression findings. We have revised the Discussion section to clarify that although sex and education were significant moderators in the univariate analyses, they were not statistically significant in the multivariable model. It has now been clearly stated that this inconsistency can be explained by collinearity among study-level characteristics, reduced statistical power due to limited number of studies, and limited variability of moderators across studies.

The following revised section has been added to the Discussion section: “Although sex and education were significantly associated with effect size in univariate meta-regression, these associations were no longer statistically significant in the multivariable model despite explaining a substantial proportion of heterogeneity. The loss of statistical significance in the multivariable meta-regression likely reflects collinearity among study-level characteristics such as sex and education, reduced statistical power due to the modest number of included studies relative to the number of moderators examined, and limited variability of several moderators across studies. These factors may have reduced the ability of the model to detect independent moderator effects despite their apparent influence in univariate analyses.”

**Moderate Comments**

**Reviewer 2 comment 7:** **Overstatement of Causality**

Statements such as: “This evidence suggests a potential causal pathway…”
Should be softened to: “This pattern is consistent with a potential causal pathway…”
Observational data cannot establish causation definitively.

**Response for Reviewer 2 comment 7:** We thank the reviewer for this valuable comment. We agree that causal interpretations should be made cautiously, given that the included studies were observational in design. Accordingly, we have revised the Discussion section to remove statements implying causality and clarified that the findings indicate significant associations rather than definitive causal relationships.

**Reviewer 2 comment 8:** **Definition Variability**

Hypertension definition: SBP ≥ 140 / DBP ≥ 90, Self-report, Medication use
ILF definition: Fixed ratio vs LLN, GOLD vs study-specific cutoffs

This heterogeneity likely affects pooled results. A dedicated paragraph discussing fixed ratio vs LLN bias would strengthen the methodological rigor.

**Response for Reviewer 2 comment 8:** We thank the reviewer for this important and constructive comment. As noted, we have previously addressed the broader issue of clinical and methodological heterogeneity in response to Reviewer 2 comment 3. In response to this additional suggestion, we have now further strengthened the Discussion section by adding a dedicated paragraph specifically addressing the potential bias associated with the use of fixed ratio versus lower limit of normal (LLN) criteria for defining impaired lung function. In addition, we have included further clarification on definition variability across studies, including differences in hypertension and variability in the classification of impaired lung function using GOLD classification versus study-specific spirometric cutoff values.

The revised text is as follows: “Although, most studies defined hypertension using the widely accepted clinical threshold of systolic blood pressure ≥ 140 mmHg and/or diastolic blood pressure ≥ 90 mmHg, some incorporated antihypertensive medication use or self-reported physician diagnosis to identify treated or previously diagnosed hypertensives, which may introduce differences in definition. Similarly, an important source of methodological variability across studies relates to differences in the definition of impaired lung function. While some studies defined impairment using the fixed ratio threshold (FEV_1_/FVC < 0.70), others applied the lower limit of normal (LLN, <5^th^ percentile) of the FEV_1_/FVC ratio based on age, sex, and population specific reference equations. These differing definitions can lead to systematic differences in case classification across studies. The fixed ratio approach does not account for the physiological decline in lung function with aging and may therefore overestimate impairment in older populations while underestimating it in younger individuals, resulting in potential age-related misclassification bias. In contrast, LLN provides individualized thresholds and reduces such misclassification by accounting for demographic variability [90]. Differences were also observed in the way airflow obstruction was classified using GOLD versus study -specific spirometric cutoff values. Because GOLD staging reflects standardized classification categories, whereas study-specific definitions may capture broader patterns of lung function impairment, these differences may affect comparability across studies and contribute to heterogeneity in pooled estimates.

**Reviewer 2 comment 9:** **Terminology Precision**

Minor but important: Use “odds ratio” not “odd ratio”
Use consistent abbreviation for impaired lung function (ILF)
Avoid redundancy in abstract (“approximately a significant one-fold higher risk” → simply “significantly higher risk”)

**Response for Reviewer 2 comment 9:** We thank the reviewer for these helpful suggestions. We have carefully revised the manuscript to improve terminology consistency and clarity. Specifically, all instances of “odd ratio” have been corrected to “odds ratio” throughout the manuscript. We have also ensured consistent use of abbreviation “impaired lung function (ILF)” following its first definition. Regarding the phrasing noted by the reviewer, we confirm that the redundancy was not present in the abstract but appeared in the Results section of the manuscript. This wording has now been revised to improve clarity and precision.

**Reviewer 2 Minor Comments:**

Remove repetition in Discussion about confounders.
Consider reducing extensive funnel plot narration; condense.
Add a summary table comparing ILF → HTN vs HTN → ILF for clarity.
Correct grammar inconsistencies throughout (copy-edit recommended).

**Response for Reviewer 2 Minor Comments:** In response to reviewer’s comment, we have revised the manuscript accordingly. Repetitive descriptions related to confounders in the Discussion section has been revised to improve clarity.

The Discussion section in the original manuscript has been revised as follows: “The pooled odds ratio remained significant in several subgroup analyses when impaired lung function was considered as the exposure. However, many subgroup categories included only a single study, limiting the ability to draw firm conclusions and requiring cautious interpretation of these findings.”

The limitations under the Discussion section in the original manuscript has been revised as follows: “Important comorbidities such as smoking burden, obesity, underlying respiratory conditions and metabolic syndrome as well as important confounders such as level of physical activity, occupational or environmental exposures, and the effect of medication were not consistently reported across studies. As a result, comprehensive adjustment for these factors was not possible, and residual confounding cannot be excluded, limiting their inclusion in subgroup or meta regression analyses.”

We have also condensed the extensive funnel plot narration. In addition, a summary table (Table 7) comparing the bidirectional associations between impaired lung function and hypertension has been added to enhance the clarity of the manuscript and facilitate comparison of key pooled findings.

**Table 7. Summary of bidirectional associations between impaired lung function and hypertension.**

| Direction of the Association | **Crude Analysis** | | | | **Adjusted Analysis** | | | |
| --- | --- | --- | --- | --- | --- | --- | --- | --- |
|  | No of studies | Sample size | OR  [95% CI] | I^2^ (%) | No of studies | Sample size | OR  [95% CI] | I^2^ (%) |
| ILF 🡪 HT | 31 | 192055 | 1.6957  [1.5312 – 1.8779] | 84.2 | 86 | 181371 | 1.3986  [1.3107 – 1.4925] | 59.8 |
| HT 🡪 ILF | 14 | 13740 | 2.9960  [1.8637 – 4.8161] | 84.7 | 6 | 10355 | 1.9417  [1.5110 – 2.4953] | 0.0 |

**Abbreviations:** ILF: Impaired lung function; HT: Hypertension; OR: Odds Ratio; I^2^: residual heterogeneity/ unaccounted variability

The manuscript has been carefully copy-edited to address grammatical inconsistencies throughout.

**Reviewer 2 Strengths of the study**

First comprehensive bidirectional meta-analysis.
Large aggregated sample size.
Inclusion of multiple continents.
Robust sensitivity analyses.
Careful quality assessment (NOS & JBI).
Attempted meta-regression.
Registration on PROSPERO.
Trim-and-fill implementation.

**Response for Reviewer 2 comment on strengths of the study:** We thank the reviewer for this helpful suggestion. In response, we have revised the strengths section to more clearly highlight key methodological strengths, including PROSPERO registration, inclusion of studies from multiple continents, meta-regression analyses, and the use of trim-and-fill methods to assess potential publication bias.

**Reviewer 2 Clinical and Public Health Significance**

This study suggests:
Lung function impairment may be an early cardiovascular risk marker.
Restrictive physiology deserves attention in cardiovascular screening.
Shared inflammatory and metabolic pathways may underlie both conditions.

For clinicians:
Patients with restrictive spirometry patterns may warrant BP monitoring.
Hypertensive patients may benefit from periodic spirometry.

**Response for Reviewer 2 comment on clinical and public health significance:** We thank the reviewer for this valuable comment on clinical and public health significance. In response, we have added the following statement in the Conclusion highlighting the clinical implications of the findings: “These findings further suggest that lung function impairment, particularly restrictive patterns, may represent an early marker of cardiovascular risk and warrant greater attention in cardiovascular screening. The observed bidirectional association also supports the possibility of shared inflammatory and metabolic mechanisms underlying both conditions. From clinical perspective, individuals with restrictive spirometry patterns may benefit form closer blood pressure monitoring, whole patients with hypertension may warrant periodic spirometry to assess lung function.”

We believe that the revised manuscript has appropriately addressed all comments raised by the reviewers. We are grateful for the opportunity to revise and resubmit our manuscript with the revisions. We hope that the revised version will now be suitable for publication.

Thank you for your continued consideration of our manuscript.

Yours sincerely,

M.L. Dilakshi Lekamge

[On behalf of all co-authors]
